# A Streamlined System for Species Diagnosis in *Caenorhabditis* (Nematoda: Rhabditidae) with Name Designations for 15 Distinct Biological Species

**DOI:** 10.1371/journal.pone.0094723

**Published:** 2014-04-11

**Authors:** Marie-Anne Félix, Christian Braendle, Asher D. Cutter

**Affiliations:** 1 Ecole Normale Supérieure, Institut de Biologie de l'ENS (IBENS), Paris, France; 2 CNRS UMR 8197, Paris, France; 3 Inserm U1024, Paris, France; 4 Institut de Biologie Valrose, CNRS UMR7277, Parc Valrose, Nice, France; 5 INSERM U1091, Nice, France; 6 Université Nice Sophia Antipolis, UFR Sciences, Nice, France; 7 Department of Ecology and Evolutionary Biology, University of Toronto, Toronto, Ontario, Canada; University of North Carolina at Chapel Hill, United States of America

## Abstract

The rapid pace of species discovery outstrips the rate of species description in many taxa. This problem is especially acute for *Caenorhabditis* nematodes, where the naming of distinct species would greatly improve their visibility and usage for biological research, given the thousands of scientists studying *Caenorhabditis*. Species description and naming has been hampered in *Caenorhabditi*s, in part due to the presence of morphologically cryptic species despite complete biological reproductive isolation and often enormous molecular divergence. With the aim of expediting species designations, here we propose and apply a revised framework for species diagnosis and description in this group. Our solution prioritizes reproductive isolation over traditional morphological characters as the key feature in delineating and diagnosing new species, reflecting both practical considerations and conceptual justifications. DNA sequence divergence criteria help prioritize crosses for establishing patterns of reproductive isolation among the many species of *Caenorhabditis* known to science, such as with the ribosomal internal transcribed spacer-2 (ITS2) DNA barcode. By adopting this approach, we provide new species name designations for 15 distinct biological species, thus increasing the number of named *Caenorhabditis* species in laboratory culture by nearly 3-fold. We anticipate that the improved accessibility of these species to the research community will expand the opportunities for study and accelerate our understanding of diverse biological phenomena.

## Introduction

Discovery of new species of *Caenorhabditis* nematode roundworms has surged in recent years, with half of the known species diversity of this genus having come to light in only the last decade [1]. This ability and urge to find new species has emerged from the improved understanding of *Caenorhabditis* natural history [2], an expanding research base in evolutionary biology of this group [3], and the growing importance of comparative analysis in molecular genetics and development among the thousands of scientists who study the model organism *C. elegans*. Unfortunately, formal species descriptions have not been able to keep pace with species discovery and with the experimental research that makes use of the new species. Only eight species enjoy Latin binomials of the nearly 30 known *Caenorhabditis* in laboratory culture [1]. Detailed experiments and comparative analyses that enlighten the biology of many ‘undescribed’ *Caenorhabditis* species are on the rise (Table S1 in File S1), and this trend will only intensify with time. The use of numerical identifiers to qualify a species is practical in the short-term but confusing when conventions change (e.g. [4] versus [5]) and thus cannot be a long-term solution. Here we propose and adopt a rationale for species determination and naming in this intensely-studied group with the aim of facilitating further *Caenorhabditis* research.

What is a species? There are many ways to answer this question, yet little universal consensus [6], excepting the generally agreed notion that species represent segments of separately evolving metapopulation lineages [7]. Historically, systematists have preferred typological and phylogenetic species concepts for delimitation, whereas experimentalists tend to favor the biological species concept [6]. Phylogenetic species concepts typically emphasize monophyly and the diagnosability of species in terms of qualitative, fixed character differences [6], [7]. By contrast, reproductive isolation between individuals provides the key feature of biological species [6], which can of course be included among the operational criteria in taxonomic species discrimination. Current views often emphasize practical or pluralistic approaches tailored to a given taxonomic group [8], or encourage the creative integration of different lines of evidence to bolster support for the existence of separately evolving lineages in species delimitation [7].

Species diagnosis depends on having identified distinguishing characteristics of organisms. Often morphological characters are used for this purpose, but any suitable feature may be used [8]. In nematode systematics, as for many other groups, morphologically cryptic species are a long-standing problem in species diagnosis [9], [10]. Indeed, Sites & Marshall [8] recommend “an eclectic approach to delimiting species and caution against the reliance on any single data set or method when delimiting species.” De Queiroz [7] reinforces this sentiment: “any property that provides evidence of lineage separation is relevant to inferring the boundaries and numbers of species…any line of evidence can be misleading if interpreted inappropriately.” With this in mind, we re-calibrate the relevant methodology for delineating and diagnosing species in *Caenorhabditis* [11]. In so doing, we reject the prerogative of the morphological description for diagnosis and naming of new *Caenorhabditis* species in favor of an operational definition emphasizing reproductive isolation, which is simpler to implement in practice and better motivated biologically to diagnose species. Both morphological and molecular criteria do, however, help to focus species diagnosis with experimental crosses and provide supporting evidence of species distinctiveness.

## Nomenclatural Acts

The electronic edition of this article conforms to the requirements of the amended International Code of Zoological Nomenclature, and hence the new names contained herein are available under that Code from the electronic edition of this article. This published work and the nomenclatural acts it contains have been registered in ZooBank, the online registration system for the ICZN. The ZooBank LSIDs (Life Science Identifiers) can be resolved and the associated information viewed through any standard web browser by appending the LSID to the prefix “ttp://zoobank.org/” The LSID for this publication is: urn:lsid:zoobank.org:pub:795AB092-12C8-4B94-BCB6-52163E832DFE. The electronic edition of this work was published in a journal with an ISSN.

### Problems with morphological type descriptions in *Caenorhabditis*

Type specimens are a cornerstone of traditional morphological systematics. Traditional species description relies on examination of morphology of type specimens and their deposition in museum collections. For example, traditional *Caenorhabditis* species descriptions are based on i) a morphological description including a set of body measurements, shape characteristics, drawings using microscopic observation and, more recently, micrographs using Nomarski optics and/or scanning electron microscopy, and ii) the storage of fixed and mounted slides of type specimens in museums. Both of these points are problematic for determining the species status of subsequent collections of *Caenorhabditis*.

First, morphological species description in *Caenorhabditis* is potentially misleading with respect to inferring the independent evolution of distinct lineages. Indeed, different species may be morphologically similar in the extreme. As an example, one of the most recent species descriptions reported “an almost complete absence of morphological differences” to distinguish *C. brenneri* from *C. remanei* with traditional taxonomic characters used in nematode systematics [12]. Kiontke et al. [1] thus noted that “Morphology can be used to assign species to the major groups within *Caenorhabditis*, but some species within these groups look very similar or entirely alike. In fact, the genus contains a host of morphological sibling species. Therefore, morphology alone is not suitable for identifying new species.” Morphological characters led to confusion and the erroneous new species description of a *C. remanei* strain as *C. vulgaris* [13], because this *C. remanei* strain did not cross with a strain of then-undescribed *C. brenneri* that had been wrongly identified as *C. remanei* based on morphological criteria [12]. The problem of morphologically similar, cryptic species also is well-known for many other groups of nematodes [9], [14]–[8].

Conversely, intraspecific variation, whether of genetic or environmental origin, may alter the morphological traits that are used for species diagnosis. For example, measurements of body length and width are polymorphic within a species due to genetic variation [19], [20] as well as being highly sensitive to age [19], [21], environmental parameters such as nutrition, pathogens and temperature [20], [22], and methodological details such as buffer and fixation solutions. Some morphological features might be expected to be more diagnostic in nematodes given the potential for strong morphological conservation, but they often prove not to be immune to these problems. For example, the overall shape and the presence or absence of a small spike of the female tail was found to vary among the progeny of a single *Cephalobus persegnis* female and, most strikingly, the presence or absence of complex lip protuberances (probolae) that were thought to be diagnostic of different genera also varied among her progeny [23]. In the *Caenorhabditis* genus, the number, position and morphology of sensory organs in the male tail provide traditional characters used for species description. Yet, these male tail traits vary within or among populations of a given species, such as *C. briggsae*, thus defying morphological species definitions [24]. While the discussion of this general problem in nematode taxonomy is not new (e.g. [23]), such morphological measurements used to infer species identity continue to be commonplace. Echoing de Queiroz [7], our opposition to the predominant use of morphological characterization for species definition in *Caenorhabditis* reflects problems of species delimitation due to the unreliability of this methodology for inferring lineage separation. Additional problems arising from such classical nematode systematics include the technically imposing morphological description, a limited access to highly specialized journals, language and alphabet barriers, nematological jargon, and lack of funding for this type of work. We do not impugn the importance of morphological quantification in studying character evolution and organismal biology; on the contrary, we advocate for more research on *Caenorhabditis* morphological evolution. However, for species descriptions in *Caenorhabditis*, we argue that these classical measurements can be counterproductive and, moreover, are neither necessary nor sufficient.

Second, deposition of fixed nematode type specimens in museums only allows interrogation of the morphology of a few individuals at a single time point. Consequently, such specimens are not useful for defining whether a newly isolated population belongs to a previously described species, because neither crosses nor DNA sequencing can be performed with the type specimen itself. Furthermore, many traits important for classical species discrimination cannot be measured on fixed and mounted slide specimens, including some morphological features but also development, life history and behavior. These limitations are especially acute in *Caenorhabditis*, for which many species descriptions are too brief to provide a useful basis for comparison to extant specimens. Several of these studies likely correspond to re-descriptions of the same species, partly owing to the difficulties of highly conserved morphology (Table S2 in File S1).

Consequently, a large fraction of named *Caenorhabditis* species are considered dubious: out of the twelve described species that are unavailable in culture, six are considered dubious and six are “morphologically well-described” [5] (Table S2 in File S1). Unfortunately, the lack of live cultures or DNA samples from these ‘well-described’ species makes it difficult or impossible to determine with certainty whether subsequently collected specimens correspond to them. How could one ever be sure that a new isolate belongs to one of these species, based on morphological description and fixed specimens?

These difficulties in morphology-based species description for *Caenorhabditis* occur in spite of enormous molecular divergence between species pairs and despite complete post-zygotic reproductive isolation between morphologically similar species. These properties hold true between *C. brenneri* and *C. remanei*, and for many other species comparisons. Conveniently, however, these characteristics of extensive sequence divergence and clear-cut reproductive isolation provide a ready means to accelerate *Caenorhabditis* species identification and naming. Moreover, there is ample historical precedent for using non-morphological features for species descriptions in general [25] and also specifically for *Caenorhabditis* [12], [26]. Together, these issues motivate and justify an approach for *Caenorhabditis* species diagnosis and description based on reproductive isolation, DNA sequence characteristics, and cryopreservation of voucher specimens. We anticipate that this approach can aid other animal groups with morphologically cryptic species, including other nematodes like *Oscheius* [27].

### A simplified protocol for *Caenorhabditis* species description based on genetic crosses, preservation of live cultures and DNA sequence analysis

A powerful aid in species delimitation in *Caenorhabditis* is the common practice among researchers to perform genetic crosses of newly isolated specimens with known species. *Caenorhabditis* is probably one of the few organisms for which the biological species concept via strong post-zygotic reproductive isolation is actually used in practice as a widespread method for species delimitation. Intrinsic post-zygotic reproductive isolation is the gold standard for species diagnosis in *Caenorhabditis*. Recent findings of F2 hybrid breakdown have motivated extension of simple ‘mating tests’ to quantitative analysis of crosses across multiple generations [28]. Such cross analysis is straightforward in *Caenorhabditis*, given the rapid generation time (2–7 days, depending on species and culture conditions) and simple rearing. Crosses have historical precedent for being used to define species in the *Caenorhabditis* genus [12], [26]. In describing *C. brenneri*, crosses and molecular distance provided the key evidence: “We conclude that *C. brenneri* sp. n. and *C. remanei* are two different species based on evidence from cross-breeding experiments, biogeography and DNA sequences, even though we have found almost no differences in morphology, physiology or ecology” [12].

**Figure 1:**
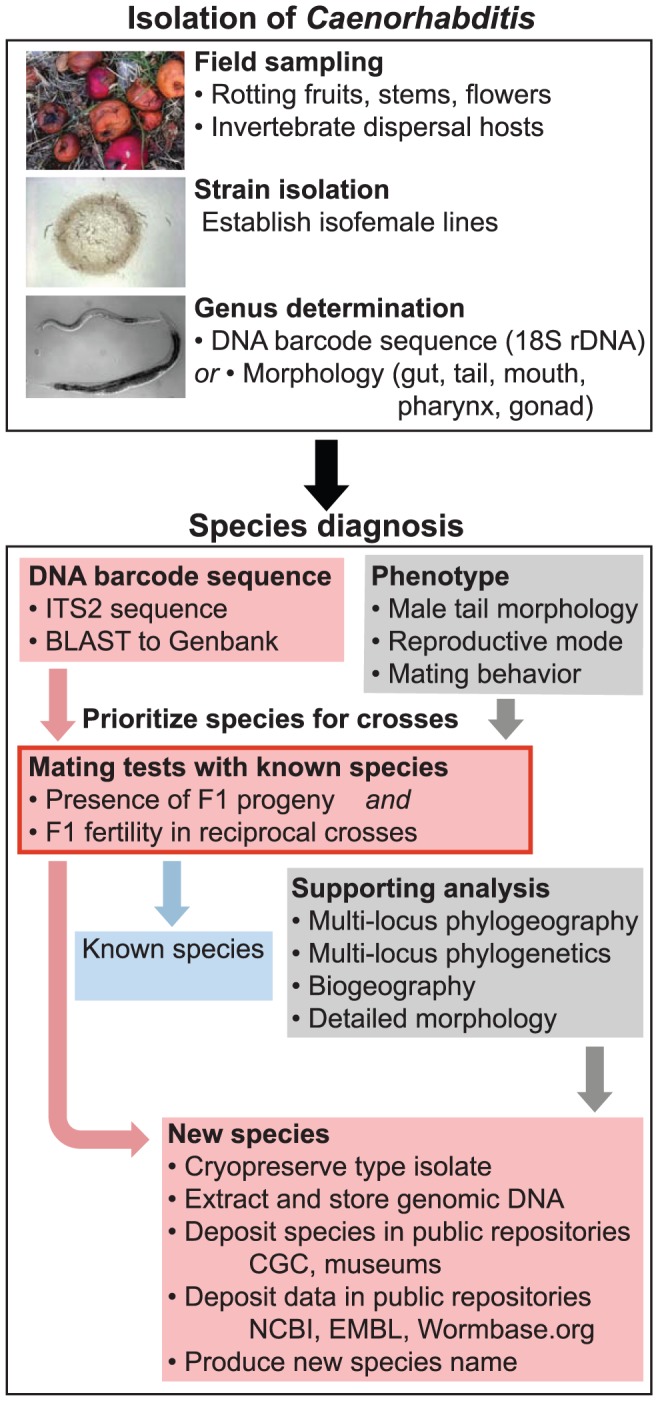
A protocol for *Caenorhabditis* species diagnosis, storage and information. *Caenorhabditis* nematodes are commonly sampled from microbe-rich habitats, such as rotting fruits [1], and cultured after isolation on standard agar plates [55]. Selection of nematodes belonging to the *Caenorhabditis* genus occurs through sequence analysis using nematode-specific primers amplifying 18S and/or through morphological criteria [55]. The species diagnosis is centered on the mating tests with known species. Due to the large number of present *Caenorhabditis* species, crosses can be best prioritized using the ITS2 barcode, and possibly phenotypic characters. A positive mating test will designate the new strain as a known species (blue). Else reproductive isolation with the closest species by ITS2 barcode, including possible isolation in reciprocal F1 crosses and backcrosses, indicates that the strain represents a new species. A suspected new species may then analyzed in more detail (gray) through multi-locus phylogenetic analysis [1]. If a novel species status can be confirmed, naming can follow immediately, and frozen live specimens as well as any relevant species information (DNA sequences, sampling details, etc.) are deposited at public repositories. The pink path summarizes the key aspects for establishing a new *Caenorhabditis* species identity based on genetic crosses with known species, prioritized from the DNA barcode results [1].

We argue that reproductive isolation among *Caenorhabditis* isolates represents a superior criterion, in theory and in practice, for species delineation in this group (Figure 1). Genetic crosses should be best supplemented by phylogenetic and/or phylogeographic analysis of sequence data from multiple nuclear loci to test for phylogenetic position and for genetic exchange among individuals and collection sites [1], [28]. Population genetic evidence of recombination among individuals can provide support for the hypothesis that the individuals derive from the same species. Biogeographic range distributions, ecological information, morphological characteristics, and behavior can provide further supporting evidence for species differences. In practice, the abundance of known biological species of *Caenorhabditis* now makes it cumbersome to genetically cross a newly-collected isolate to all possible species. Thus, application of an ITS2 (ribosomal rDNA internal transcribed spacer-2) sequence barcode helps to prioritize crosses to those species with the most similar ITS2 sequence, whereas it is not adequate for purposes of phylogenetic reconstruction [1]. The classical molecular markers 18S and 28S rDNA are generally adequate for genus identification but provide insufficient phylogenetic resolution within *Caenorhabditis* to help prioritize crosses [1], [29]. Note that we do not advocate use of the ITS2 sequence as an absolute criterion for species diagnosis, but only to prioritize crosses of a new isolate with known species. Indeed, ITS2 is polymorphic within some species and, conversely, some distinct biological species could potentially share identical ITS2 sequence. In sum, as pioneered in [1], we thus advocate the use of the ITS2 sequence barcode, and any other phylogenetically informative genes with widespread representation in sequence databases (e.g. those in [1]), to help prioritize crosses. Because of this utility, we strongly recommend that species naming articles include this molecular sequence information, or a reference to published work providing it, so as to ease future characterization of new isolates.

As explained above, reliance on traditional fixed type specimens as a reference for the species name should be relaxed. Two key features of *Caenorhabditis* make alternatives practical. First, standard practice in collection of wild isolates involves establishing iso-female lines (i.e. strains founded by a single gravid female or hermaphrodite) which are assigned unique identifiers based on the established, universal strain naming scheme used world-wide by *C. elegans* researchers [30]. The use of iso-female lines makes sure that the culture does not contain several species from the original sample. Second, nearly all species of *Caenorhabditis* known to date can be easily cryopreserved with standard protocols [30]. Thousands of genetically unique strains of *C. elegans* and other *Caenorhabditis* species are thus stored at the Caenorhabditis Genetics Center repository, and thousands of additional wild isolates are stored in -80°C freezers and liquid nitrogen storage units of independent research labs, which can be thawed, regrown and shared as needed among researchers. Such cryopreserved strains can be stored indefinitely, thawed and cultured when needed for any further studies, including genome sequence and phenotypic characterization. This protocol can therefore effectively substitute traditional protocols, obviating the need to store single, dead specimens. We propose that cryopreserved living voucher specimens provide a superior implementation of type specimen preservation for *Caenorhabditis* nematodes. All newly identified *Caenorhabditis* species should be preserved in public repositories for scientific research, such as the Caenorhabditis Genetics Center (http://www.cgc.cbs.umn.edu/), in addition to research labs. Natural history museum curation of diverse species collections continues to be an essential institution, and therefore should extend curation to cryogenic archiving of organisms like *Caenorhabditis*.

Here we summarize a minimum framework required for species designation of new *Caenorhabditis* (Figure 1): i) genetic crosses to close relatives indicating reproductive isolation; ii) a reference (type) isolate, representing an isofemale line that is cryopreserved in public repositories; iii) an ITS2 tag sequence and/or molecular phylogeographic analysis in the case of very close species pairs [28]. Additional characterizations of divergence in morphology, behavior, geography, or ecology can provide valuable supporting evidence; traditional voucher specimens fixed and mounted on slides for museum collections also are encouraged. For some species that are recalcitrant to cryopreservation, growing cultures, traditional fixed and mounted slide specimens, and storage of ample quantities of DNA may be necessary alternatives in these cases.

### Species diagnosis and naming for 15 species of *Caenorhabditis*

**Figure 2:**
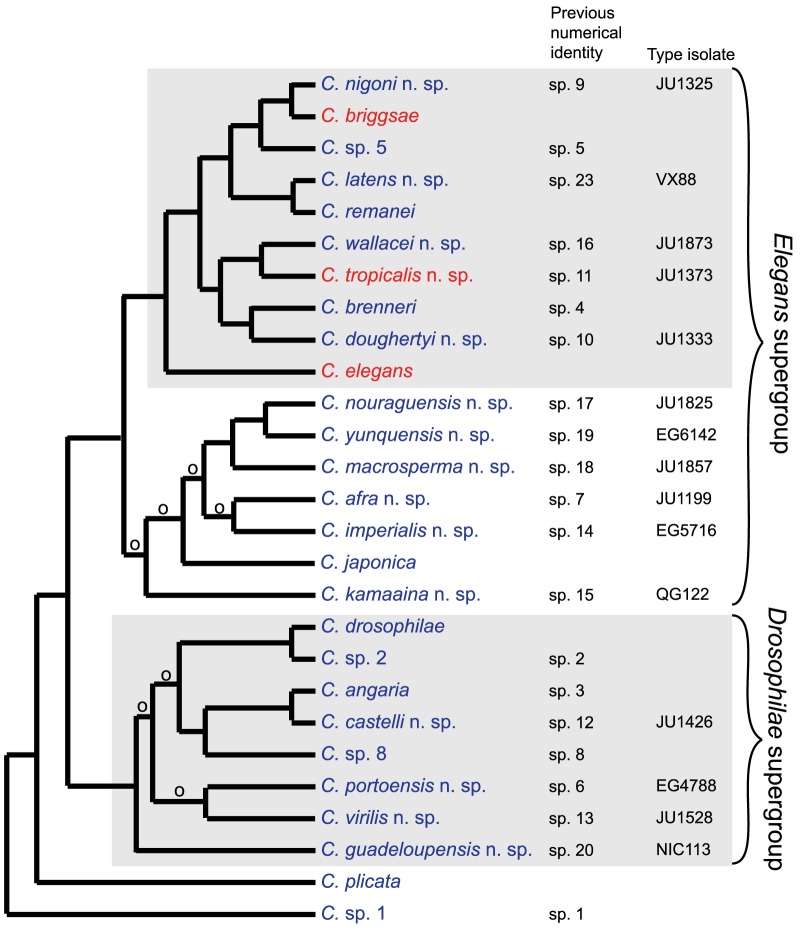
Phylogenetic topology of named species of *Caenorhabditis* in laboratory culture. Androdioecious species with hermaphrodites are indicated in red; gonochoristic species with females are indicated in blue. The *Elegans* group and *Drosophilae* supergroup are highlighted with gray background. Distant outgroup *Pristionchus pacificus* is indicated in gray text. Cladogram is redrawn from [1], where ‘o’ indicates branches with low support.

We apply the above rationale to provide Latin binomials for each of 15 distinct species of *Caenorhabditis* that meet the criteria of biological reproductive isolation and molecular phylogenetic distinctiveness. Consistent with previous literature, *Caenorhabditis* sp. 1 currently represents the most external recognized lineage in the genus [1], [5], [31]. Note that *Caenorhabditis* sp. 1 and a few other ‘numbered’ species are not named here, because they are currently being formally described by others. *C. elegans* was the first species described of the genus [1]. The 15 species given below are ingroups compared to *Caenorhabditis* sp. 1 and *Caenorhabditis plicata* and therefore species of *Caenorhabditis* [1], [5] (Figure 2). Crossing and sequence data are unavailable for the species listed in Table S2 in File S1, of which six have been deemed dubious [5]. Therefore, we conclude that it is impossible to determine potential synonymy of fixed specimen with these new species. Below are the diagnostic features of each species to which we apply a name. For each species, we refer explicitely to publicly available data concerning cross results and their ribosomal SSU, LSU and ITS2 sequences from crosses that allow delineation as separate species and diagnosis of new isolates.

*Caenorhabditis portoensis* Félix, Braendle & Cutter sp. nov.

urn:lsid:zoobank.org:act:F473841A-9BDC-4FE1-B00B-20FC777489C4

 =  Caenorhabditis sp. 6 in [1]

The type isolate by present designation is EG4788. The species is delineated and diagnosed by the positive result of the cross with the type isolate EG4788 in both cross directions, yielding highly fertile hybrid females and males that are interfertile and cross-fertile with their parent strains. The species reproduces through males and females [1]. This species differs by SSU, LSU and ITS2 DNA sequences (JN636066) from all other species in [1], listed in Tables 1 and 2. Note that these ribosomal DNA sequences may vary within the species. A single isolate for this species is known so far, from Amares, Portugal [1]. *C. portoensis* n. sp. does not belong to the *Elegans* super-group, as indicated by molecular and morphological characters [1], and does not morphologically resemble *C. sonorae* [1], [32]. See [1] for a drawing of male genitalia.

**Table 1:** New species name designations for *Caenorhabditis*.

New species name	Previous species number	Type isolate	Abbreviation for genetic nomenclature
*Caenorhabditis portoensis* n. sp.	*C*. sp. 6	EG4788	*Cpo*
*Caenorhabditis afra* n. sp.	*C*. sp. 7	JU1199	*Caf*
*Caenorhabditis nigoni* n. sp.	*C*. sp. 9	JU1325	*Cni*
*Caenorhabditis doughertyi* n. sp.	*C*. sp. 10	JU1333	*Cdo*
*Caenorhabditis tropicalis* n. sp.	*C*. sp. 11	JU1373	*Ctr*
*Caenorhabditis castelli* n. sp.	*C*. sp. 12	JU1426	*Cca*
*Caenorhabditis virilis* n. sp.	*C*. sp. 13	JU1528	*Cvi*
*Caenorhabditis imperialis* n. sp.	*C*. sp. 14	EG5716	*Cim*
*Caenorhabditis kamaaina* n. sp.	*C*. sp. 15	QG122	*Cka*
*Caenorhabditis wallacei* n. sp.	*C*. sp. 16	JU1873	*Cwa*
*Caenorhabditis nouraguensis* n. sp.	*C*. sp. 17	JU1825	*Cno*
*Caenorhabditis macrosperma* n. sp.	*C*. sp. 18	JU1857	*Cma*
*Caenorhabditis yunquensis* n. sp.	*C*. sp. 19	EG6142	*Cyu*
*Caenorhabditis guadeloupensis* n. sp.	*C*. sp. 20	NIC113	*Cgu*
*Caenorhabditis latens* n. sp.	*C*. sp. 23	VX88	*Cla*

6: found near Porto; 7: found in West Africa; 9: in honor of Victor Marc Nigon, pioneer in the use of *C. elegans* in biology, co-describer of *C. briggsae*; 10: in honor of Ellsworth Dougherty, pioneer in the use of *Caenorhabditis* in biology, co-describer of *C. briggsae*; 11: tropical distribution; 12: in honor of Patrick Châtelet who collected the sample at the CNRS Nouragues station, the small castle over which he reigns; 13: with a remarkable male tail; 14: imperial; 15: from Hawaii, in Hawaiian language; 16: in honor of Alfred Wallace and Indonesian biogeography; 17: found in the Natural Reserve of the Nouragues, French Guiana; 18: exhibits very large male sperm [1]; 19: found in El Yunque; 20: found in Guadeloupe; 23: previously hidden.

**Table 2:** Other named species of *Caenorhabditis* in laboratory culture.

Species name	Previous species number	Abbreviation for genetic nomenclature	Species description reference
*C. angaria*	*C*. sp. 3	*Can*	Sudhaus et al. 2011 [49]
*C. briggsae*	NA	*Cbr*	Dougherty & Nigon 1949 [50]
*C. brenneri*	*C*. sp. 4	*Cbn*	Sudhaus & Kiontke 2007 [12]
*C. drosophilae*	NA	*Cdr*	Kiontke 1997 [32]
*C. elegans*	NA	*Cel*	Maupas 1900 [51]
*C. japonica*	NA	*Cja*	Kiontke et al. 2002 [52]
*C. plicata*	NA	*Cpl*	Völk 1950 [53]
*C. remanei*	NA	*Cre*	Sudhaus 1974 [54]

*Caenorhabditis afra* Félix, Braendle & Cutter sp. nov.

urn:lsid:zoobank.org:act:E705B72C-1E5D-469B-BC6A-9E539B3FDD8D

 =  Caenorhabditis sp. 7 in [1]

The type isolate by present designation is JU1199. The species is delineated and diagnosed by the fertile cross with the type isolate JU1199 in both cross directions, yielding highly fertile hybrid females and males that are interfertile and cross-fertile with their parent strains. The species reproduces through males and females [1]. This species differs by SSU, LSU and ITS2 DNA sequences (JN636064) from all other species in [1], listed in Tables 1 and 2. Note that these ribosomal DNA sequences may vary within the species. From molecular data, the closest species is *C. imperialis* n. sp., with which it does not form any larval progeny [1]. The type isolate was collected in Begor, Ghana, and another isolate was found in Nigeria [1]. See [1] for a drawing of male genitalia.

*Caenorhabditis nigoni* Félix, Braendle & Cutter sp. nov.

urn:lsid:zoobank.org:act:2538A344-637C-434E-8A80-2A16B184A82C

 =  *Caenorhabditis* sp. 9 in [1] and articles listed in Table S1 in File S1

The type isolate by present designation is JU1325. The species is delineated and diagnosed by the fertile cross with the type isolate JU1325 in both cross directions, yielding highly fertile hybrid females and males that are interfertile and cross-fertile with their parent strains. The species reproduces through males and females [1]. This species differs by SSU, LSU and ITS2 DNA sequences (JN636060) from all other species in [1], listed in Tables 1 and 2. Note that these ribosomal DNA sequences may vary within the species. Molecular evidence shows that the androdioecious *C. briggsae* is its closest relative, a species with which it can form only partially-fertile cross-progeny [1], [33], [34]. The type isolate was collected in Trivandrum, Kerala, India and another isolate was collected in Congo-Kinshasa [1]. See [1] for a drawing of male genitalia.

*Caenorhabditis doughertyi* Félix, Braendle & Cutter sp. nov.

urn:lsid:zoobank.org:act:6D1A7C02-982E-472E-8619-F6746AB78B51

 =  *Caenorhabditis* sp. 10 in [1]

The type isolate by present designation is JU1133. The species is delineated and diagnosed by the fertile cross with the type isolate JU1133 in both cross directions, yielding highly fertile hybrid females and males that are interfertile and cross-fertile with their parent strains. The species reproduces through males and females [1]. This species differs by SSU, LSU and ITS2 DNA sequences (JN636062) from all other species in [1], listed in Tables 1 and 2. Note that these ribosomal DNA sequences may vary within the species. From molecular data, the closest species is *C. brenneri*, with which it does not form any larval progeny [1]. The type isolate was collected next to Periyar, Kerala, India and other isolates were collected in Kerala [1]. See [1] for a drawing of male genitalia.

*Caenorhabditis tropicalis* Félix, Braendle & Cutter sp. nov.

urn:lsid:zoobank.org:act:E35C180C-08A8-4C91-8F44-390A6A29456E

 =  *Caenorhabditis* sp. 11 in [1] and articles listed in Table S1 in File S1

The type isolate by present designation is JU1373. The species is delineated and diagnosed by the fertile cross with the type isolate JU1373 in both cross directions, yielding highly fertile hybrid hermaphrodites and males that are interfertile and cross-fertile with their parent strains. The species reproduces through self-fertile hermaphrodite and facultative males [1]. This species differs by SSU, LSU and ITS2 DNA sequences (JN636063) from all other species in [1], listed in Tables 1 and 2. Note that these ribosomal DNA sequences vary within the species [1]. This androdioecious species does not cross successfully with either *C. elegans* or *C. briggsae* [1]. From molecular data, the closest species is *C. wallacei* n. sp., with which it does not form viable progeny [1]. The type isolate was collected in Saint-Benoît, La Réunion and other isolates were collected in Cape Verde, Hawaii, Guadeloupe, Puerto Rico, Brazil and French Guiana [1], [35]. See [1] for a drawing of male genitalia.

*Caenorhabditis castelli* n. sp. Félix, Braendle & Cutter sp. nov.

urn:lsid:zoobank.org:act:31D1A176-8517-42A5-94C9-2A6F3EC7E710

 =  *Caenorhabditis* sp. 12 in [1]

The type isolate by present designation is JU1426. The species is delineated and diagnosed by the fertile cross with the type isolate JU1426 in both cross directions, yielding highly fertile hybrid females and males that are interfertile and cross-fertile with their parent strains. The species reproduces through males and females [1]. This species differs by SSU, LSU and ITS2 DNA sequences (JN636069) from all other species in [1], listed in Tables 1 and 2. Note that these ribosomal DNA sequences may vary within the species. From molecular data, the closest species is *C. angaria* with which it forms sterile hybrid progeny [1]. The type isolate was collected in Nouragues, French Guiana and another isolate was collected in the same location [1]. See [1] for a drawing of male genitalia.

*Caenorhabditis virilis* Félix, Braendle & Cutter sp. nov.

urn:lsid:zoobank.org:act:E35C180C-08A8-4C91-8F44-390A6A29456E

 =  *Caenorhabditis* sp. 13 in [1]

The type isolate by present designation is JU1528. The species is delineated and diagnosed by the fertile cross with the type isolate JU1528 in both cross directions, yielding highly fertile hybrid females and males that are interfertile and cross-fertile with their parent strains. The species reproduces through males and females [1]. This species differs by SSU, LSU and ITS2 DNA sequences (JN636067) from all other species in [1], listed in Tables 1 and 2. Note that these ribosomal DNA sequences may vary within the species. The species is molecularly divergent from all other species [1]. The type isolate was collected in Orsay, France and another isolate was collected in the same location [1]. See [1] for a drawing of male genitalia.

*Caenorhabditis imperialis* Félix, Braendle & Cutter sp. nov.

urn:lsid:zoobank.org:act:8B81E334-CEC6-433F-8B45-C92573092EDB

 =  *Caenorhabditis* sp. 14 in [1]

The type isolate by present designation is EG5716. The species is delineated and diagnosed by the fertile cross with the type isolate EG5716 in both cross directions, yielding highly fertile hybrid females and males that are interfertile and cross-fertile with their parent strains. The species reproduces through males and females [1]. This species differs by SSU, LSU and ITS2 DNA sequences (JN636140) from all other species in [1], listed in Tables 1 and 2. Note that these ribosomal DNA sequences may vary within the species. From molecular data, the closest species is *C. afra* n. sp., with which it does not form any larval progeny [1]. The type isolate was collected in Moorea, French Polynesia, and other isolates were collected in Guadeloupe [1]. See [1] for a drawing of male genitalia.

*Caenorhabditis kamaaina* Félix, Braendle & Cutter sp. nov.

urn:lsid:zoobank.org:act:B9DF901D-55A1-447B-B697-4000AAFE5D01

 =  *Caenorhabditis* sp. 15 in [1]

The type isolate by present designation is QG122. The species is delineated and diagnosed by the fertile cross with the type isolate QG122 in both cross directions, yielding highly fertile hybrid females and males that are interfertile and cross-fertile with their parent strains. The species reproduces through males and females [1]. This species differs by SSU, LSU and ITS2 DNA sequences (JN636141) from all other species in [1], listed in Tables 1 and 2. Note that these ribosomal DNA sequences may vary within the species. The species is distant molecularly from all other species [1]. The type isolate was collected in Kauai, Hawaii, and another isolate was collected in Kauai [1]. See [1] for a drawing of male genitalia.

*Caenorhabditis wallacei* Félix, Braendle & Cutter sp. nov.

urn:lsid:zoobank.org:act:E4470BAC-8F05-4ECC-9DC1-DC1D6D0E9741

 =  *Caenorhabditis* sp. 16 in [1]

The type isolate by present designation is JU1873. The species is delineated and diagnosed by the fertile cross with the type isolate JU1873 in both cross directions, yielding highly fertile hybrid females and males that are interfertile and cross-fertile with their parent strains. The species reproduces through males and females [1]. This species differs by SSU, LSU and ITS2 DNA sequences (JN636137) from all other species in [1], listed in Tables 1 and 2. Note that these ribosomal DNA sequences may vary within the species. From molecular data, the closest species is the selfing *C. tropicalis* n. sp. with which it does not form viable progeny [1]. The type isolate was collected in Sanda Center, Bali, Indonesia [1]. See [1] for a drawing of male genitalia.

*Caenorhabditis nouraguensis* Félix, Braendle & Cutter sp. nov.

urn:lsid:zoobank.org:act:4E16AA1C-6FFE-4098-87BC-79F1E4959086

 =  *Caenorhabditis* sp. 17 in [1]

The type isolate by present designation is JU1825. The species is delineated and diagnosed by the fertile cross with the type isolate JU1825 in both cross directions, yielding highly fertile hybrid females and males that are interfertile and cross-fertile with their parent strains. The species reproduces through males and females [1]. This species differs by SSU, LSU and ITS2 DNA sequences (JN636139) from all other species in [1], namely all other species listed in Tables 1 and 2. Note that these ribosomal DNA sequences vary within the species [1]. From molecular data, the closest species are *C. macrosperma* n. sp. and *C. yunquensis* n. sp., with which it does not form any larval progeny [1]. The type isolate was collected in Nouragues, French Guiana and other isolates were collected in French Guiana [1], [31], [36]–[39]. See [1] for a drawing of male genitalia.

*Caenorhabditis macrosperma* Félix, Braendle & Cutter sp. nov.

urn:lsid:zoobank.org:act:DB7D2F62-D33F-4EDA-B85F-0FE780C74A53

 =  *Caenorhabditis* sp. 18 in [1]

The type isolate by present designation is JU1857. The species is delineated and diagnosed by the fertile cross with the type isolate JU1857 in both cross directions, yielding highly fertile hybrid females and males that are interfertile and cross-fertile with their parent strains. The species reproduces through males and females [1]. This species differs by SSU, LSU and ITS2 DNA sequences (JN636138) from all other species in [1], namely all other species listed in Tables 1 and 2. Note that these ribosomal DNA sequences may vary within the species. From molecular data, the closest species are *C. nouraguensis* n. sp. and *C. yunquensis* n. sp., with which it does not form any larval progeny [1]. The type isolate was collected in Nouragues, French Guiana and another isolate was collected in the same location [1], [31], [36]–[39]. See [1] for a drawing of male genitalia.

*Caenorhabditis yunquensis* Félix, Braendle & Cutter sp. nov.

urn:lsid:zoobank.org:act:D060F572-EF49-479F-9074-69A8B82D57F6

 =  *Caenorhabditis* sp. 19 in [1]

The type isolate by present designation is EG6142. The species is delineated and diagnosed by the fertile cross with the type isolate EG6142 in both cross directions, yielding highly fertile hybrid females and males that are interfertile and cross-fertile with their parent strains. The species reproduces through males and females [1]. This species differs by SSU, LSU and ITS2 DNA sequences (JN636136) from all other species in [1], listed in Tables 1 and 2. Note that these ribosomal DNA sequences may vary within the species. From molecular data, the closest species are *C. macrosperma* n. sp. and *C. nouraguensis* n. sp., with which it does not form any larval progeny [1]. A single isolate of this species is known so far, from El Yunque, Puerto Rico [1]. See [1] for a drawing of male genitalia.

*Caenorhabditis guadeloupensis* Félix, Braendle & Cutter sp. nov.

urn:lsid:zoobank.org:act:DEB4B944-253F-4314-A38F-DF3B7CECE824

 =  *Caenorhabditis* sp. 20 in [1]

The type isolate by present designation is NIC113. The species is delineated and diagnosed by the fertile cross with the type isolate NIC113 in both cross directions, yielding highly fertile hybrid females and males that are interfertile and cross-fertile with their parent strains. The species reproduces through males and females [1]. This species differs by SSU, LSU and ITS2 DNA sequences (JN636135) from all other species in [1], listed in Tables 1 and 2. Note that these ribosomal DNA sequences may vary within the species. The species is molecularly distant from all other species [1]. A single isolate of this species is known so far, from the Soufrière Forest trail, Guadeloupe [1]. See [1] for a drawing of male genitalia.

*Caenorhabditis latens* Félix, Braendle & Cutter sp. nov.

urn:lsid:zoobank.org:act:C046BE47-3E23-4A7D-A760-6E1670BA3838

 =  *Caenorhabditis* sp. 23 in [28]

The type isolate by present designation is VX88. The species is delineated and diagnosed by the fertile cross with the type isolate VX88 in both cross directions, yielding hybrids that are all interfertile and cross-fertile with their parental strains. The species reproduces through males and females [1]. This species differs by SSU, LSU and ITS2 DNA sequences (JN636111) from all other species in [1], listed in Tables 1 and 2. Note that these ribosomal DNA sequences may vary within the species. The species reproduces through males and females [28]. It is closely related to *C. remanei*, with which it can make partially fertile hybrids [28]. Phylogeographic analysis of 20 nuclear protein coding genes strongly supports its genetic distinctiveness from *C. remanei* [28]. The type isolate was collected in Jiufeng Village, Wuhan City, Hubei, China and other isolates were collected in China [28].

### Additional considerations on reproductive isolation in *Caenorhabditis*

Here we emphasize reproductive isolation as a key practical, and conceptually-motivated, criterion for delimiting species of *Caenorhabditis*. Most species pairs in this genus show complete intrinsic post-zygotic reproductive isolation (i.e. no fertile hybrids result from crosses), making this criterion clear and simple to interpret. However, with accelerating efforts to discover new species, we anticipate growth in the number of species pairs that will exhibit only partial intrinsic post-zygotic isolation. For example, the cryptic *C. latens* n. sp. was identified only upon recognizing strong F2 hybrid breakdown, despite the formation of nearly normal numbers of F1 progeny, in crosses with *C. remanei* [28]. This raises the question of the magnitude of reproductive isolation required to diagnose a distinct species. The study of the speciation process in *Caenorhabditis* is still in its infancy, so far having focused on intrinsic post-zygotic isolation [28], [33], [34], [40], [41]. Similarly, the ecological context of *Caenorhabditis* nematodes is only beginning to be understood [2]. Consequently, we anticipate that reproductive isolation mediated through intrinsic pre-zygotic mechanisms (e.g. assortative mating, gametic barriers), as well as extrinsic mechanisms (i.e. environment-dependence), might also be discovered to contribute to the separation of lineages. We should therefore expect these forms of evidence for reproductive incompatibility to contribute to *Caenorhabditis* species diagnosis in the future. However, we also caution against over-interpretation and splintering of accepted species from findings such as outbreeding depression [35], [42] and single-locus incompatibility systems driven by selfish genetic elements [43], [44], especially in highly self-fertilizing species. Nevertheless, such phenomena may prove powerful in studying the speciation process itself at its earliest stages [45], when incipient species may be especially prone to heritable variability in isolating barriers [46].

A second caution applies to using molecular sequence data to guide species diagnosis. Population genetic diversity within any given species of *Caenorhabditis* can be very high, with the extreme example of *C. brenneri* showing the highest known molecular diversity among eukaryotes [47], [48]. Consequently, heuristic thresholds of sequence divergence cannot prove decisive in inferring species identity. Population genetic evidence of recombination can, however, provide supporting evidence of species membership. The most powerful role for DNA sequence information in species diagnosis is in helping to prioritize which known species to use in crosses to test for reproductive isolation.

## Conclusions

The rapid pace of new species discovery in *Caenorhabditis* nematodes, and a stream of associated primary research on such novel species, requires an accelerated means of species determination in this group. We provide such a simplified protocol for determining *Caenorhabditis* species identity, allowing rapid species naming to facilitate research of these intensely studied animals. We define reproductive isolation as the key criterion in species delimitation, complemented by molecular sequence and phylogenetic analysis. This approach thus incorporates both practical considerations and conceptually meaningful motivations for *Caenorhabditis* species descriptions. The standard procedure of cryopreserving iso-female lines of all species in public repositories allows secure storing and easy access of live specimens for researchers. Morphological, developmental and behavioral studies can then be extended to address salient biological questions in character evolution, with the ability to control for genetic and environmental sources of phenotypic variation.

By adopting this protocol for species delimitation, here we provide new species name designations for 15 species of *Caenorhabditis* (Table 1). Previous research on these species has already substantially aided our understanding of a wide range of topics, ranging from phylogeography, population genetics and speciation to genetic editing, RNA interference, and developmental biology (Table S1 in File S1). We anticipate that explicit species names will improve recognition and accessibility for research of these distinct *Caenorhabditis* species, thereby further enhancing our understanding of diverse biological phenomena.

## Supporting Information

File S1 Contains the following files: Table S1. Literature devoted to study of previously unnamed *Caenorhabditis* species. Table S2. Other named *Caenorhabditis* species. Supplementary References.
(DOCX)
