# Correction: A Streamlined System for Species Diagnosis in *Caenorhabditis* (Nematoda: Rhabditidae) with Name Designations for 15 Distinct Biological Species

**DOI:** 10.1371/journal.pone.0118327

**Published:** 2015-03-05

**Authors:** The PLOS ONE Staff

In the subheading “Species diagnosis and naming for 15 species of *Caenorhabditis*” of Nomenclatural Acts, the following sentence was omitted from the paragraphs of description for all 15 species: “The type culture specimens are deposited at the Caenorhabditis Genetics Center (http://www.cbs.umn.edu/research/resources/cgc).”
