# Supplementary material for: A Streamlined System for Species Diagnosis in Caenorhabditis (Nematoda: Rhabditidae) with Name Designations for 15 Distinct Biological Species
Source: PLoS One. 2014 Apr 11;9(4):e94723. doi: 10.1371/journal.pone.0094723 (PMC3984244; doi:10.1371/journal.pone.0094723)
Supplement: File S1 — Contains the following files: Table S1. Literature devoted to study of previously unnamed Caenorhabditis species. Table S2. Other named Caenorhabditis species. Supplementary References. (DOCX) [file pone.0094723.s001.docx]

**Table S1. Literature devoted to study of previously unnamed *Caenorhabditis* species.**

| Species name | Species number | References |
| --- | --- | --- |
| *Caenorhabditis* sp. 2 | *C*. sp. 2 | [[1](#_ENREF_1)] |
| *Caenorhabditis* sp. 5 | *C*. sp. 5 | [[1-6](#_ENREF_1)] |
| *Caenorhabditis nigoni* n. sp. | *C*. sp. 9 | [[7-12](#_ENREF_7)] |
| *Caenorhabditis tropicalis* n. sp. | *C*. sp. 11 | [[13](#_ENREF_13),[14](#_ENREF_14)] |
| *Caenorhabditis latens* n. sp. | *C*. sp. 23 | [[15](#_ENREF_15),[16](#_ENREF_16)] |
| Multiple species | Multiple | [[17-27](#_ENREF_17)] |

**Table S2. Other named *Caenorhabditis* species** [[17](#_ENREF_17),[28](#_ENREF_28)].

| Species name | Species description reference | Likely reproductive mode | Status in [[17](#_ENREF_17)] | Comments |
| --- | --- | --- | --- | --- |
| *anthobia* | [Schneider 1937](#_ENREF_43) [[29](#_ENREF_29)] | Unclear | Found once,  Indonesia | Schneider [[29](#_ENREF_29)] only provides a very brief morphological description based on 10 females and one male. Ray 3 was overlooked [[29](#_ENREF_29)]. The species is described to be viviparous (under unknown environmental conditions), unlike any of the species named here in standard *C. elegans* laboratory culture conditions. Schneider only compares this species with "Rhabditis" species that are now considered outside *Caenorhabditis* (thus not even *C. elegans*), namely species now called *Oscheius dolichura* and *Buetschlinema pellioides* [[28](#_ENREF_28)]. |
| *auriculariae* | Tsuda & Futai 1999 [[30](#_ENREF_30)] | F | Found once, recent years,  Japan | Morphological characters such as the the anteriorly closed male fan placed this species in the *Elegans* group in [[28](#_ENREF_28)], yet this character was found to be plesiomorphic in [[29](#_ENREF_29)]. The shape of the pre-cloacal lip would instead place it outside the *Elegans* super-group in [[29](#_ENREF_29)]. Dorsal opening of rays 5+8 is not found in any of the 15 species described here [[29](#_ENREF_29)]. |
| *avicola* | Schmidt & Kuntz 1972 [[31](#_ENREF_31)] | F | Found once,  Taiwan | Figure 3 in [[31](#_ENREF_31)] only provides a schematic drawing of male tail; dorso-ventral positions of ray openings are not indicated, which makes it difficult to compare. The anterior end of the fan is pointed, unlike that of any species named here [[29](#_ENREF_29)], yet this may be due to folds. |
| *bovis* | Kreis 1964 [[32](#_ENREF_32)] | Unclear | Found regularly in bovine ears (East Africa) | Male tail shape and ray configuration on the drawing [[32](#_ENREF_32)] do not resemble any other species, including the 15 named in this work [[29](#_ENREF_29)]. Distal female ovary does not appear to be syncytial. The species is described to be viviparous (under unknown environmental conditions), unlike any of the species named here in standard *C. elegans* laboratory culture conditions. |
| *clavopapillata* | Kreis & Faust 1933 [[33](#_ENREF_33)] | Unclear | Dubious | The description mentions only 8 rays in the male tail, unlike any of the species named here, which all have 9 rays [[29](#_ENREF_29)], except for variation in *C. briggsae* (8 or 9) [[34](#_ENREF_34)]. |
| *craspedocerca* | Völk 1950 [[35](#_ENREF_35)] | H | Dubious | Völk [[35](#_ENREF_35)] only provides a very short description, with no drawing of the male tail and no comparison with previous species such as *C. elegans, C. briggsae* and *C. perrieri*. Males are rare. |
| *formosana* | Yokoo & Okabe 1968 [[36](#_ENREF_36)] | F | Dubious | Male tail shape and ray configuration on the drawing [[32](#_ENREF_32)] do not resemble any other species, including the 15 named in this work [[29](#_ENREF_29)]. The anterior end of fan seems anteriorly closed on a picture, yet not on the drawing. |
| *fruticicolae* | Shinohara 1960 [[37](#_ENREF_37)] | F | Dubious | The description mentions 10 rays in the male tail, unlike the species named here, which all have 9 rays [[29](#_ENREF_29)]. |
| *genitalis* | Scheiber 1880 [[38](#_ENREF_38)] | F | Dubious | The drawings are rudimentary. The posterior end of the male fan seems pointed, unlike that of any species named here [[29](#_ENREF_29)] |
| *oncomelaniae* | Yokoo & Okabe 1968 [[36](#_ENREF_36)] | F | Dubious | The male tail of this species resembles that of *Elegans* super-group species [[29](#_ENREF_29)]. The description only compares this species with the other species that has been previously found in *Oncomelania* snails, which is not in the *Caenorhabditis* genus [[36](#_ENREF_36)]. |
| *perrieri* | Maupas 1900 [[39](#_ENREF_39)] | H | Found once,  Algeria | The description does not indicate the dorso-ventral positions of ray openings [[39](#_ENREF_39)]. The arrangement of male rays 1-3, with ray 3 close to rays 1-2, differs from that of *C. elegans* and *C. briggsae* and does not resemble either that of *C*. *tropicalis* n. sp. [[29](#_ENREF_29)]. It cannot be ruled out that this was a morphological variant of one of the selfing species presently in culture. |
| *sonorae* | Kiontke 1997 [[40](#_ENREF_40)] | F | Found once, recent years,  Arizona | The male tail of *C.* sp. 1 [[29](#_ENREF_29)] resembles that of *C. sonorae*, but the former species is monodelphic (single fertile ovary arm), unlike the latter. The relatedness of these two species is represented in [[29](#_ENREF_29)]. |

F: male-female. H: hermaphrodite with facultative males. *based on morphological characters such as anteriorly closed fan, found likely to be plesiomorphic by [[19](#_ENREF_19)]. The male bursa in classical descriptions corresponds to the fan in *C. elegans* nomenclature, and the rays to caudal papillae, numbered 1 to 9 from anterior to posterior.

**Supplementary References**

1. Félix M-A (2007) Cryptic quantitative evolution of the vulva intercellular signaling network in *Caenorhabditis*. Current Biology 17: 103-114.

2. Cutter AD (2008) Divergence times in *Caenorhabditis* and *Drosophila* inferred from direct estimates of the neutral mutation rate. Molecular Biology and Evolution 25: 778-786.

3. Cutter AD, Wasmuth JD, Washington NL (2008) Patterns of molecular evolution in Caenorhabditis preclude ancient origins of selfing. Genetics 178: 2093-2104.

4. Cutter AD, Wang G-X, Ai H, Peng Y (2012) Influence of finite-sites mutation, population subdivision and sampling schemes on patterns of nucleotide polymorphism for species with molecular hyperdiversity. Molecular Ecology 21: 1345-1359.

5. Wang G-X, Ren S, Ren Y, Ai H, Cutter AD (2010) Extremely high molecular diversity within the East Asian nematode *Caenorhabditis* sp. 5. Molecular Ecology 19: 5022-5029.

6. Jovelin R, Cutter AD (2011) MicroRNA sequence variation potentially contributes to within-species functional divergence in the nematode *Caenorhabditis briggsae*. Genetics 189: 967-976.

7. Woodruff GC, Eke O, Baird SE, Félix MA, Haag ES (2010) Insights into species divergence and the evolution of hermaphroditism from fertile interspecies hybrids of Caenorhabditis nematodes. Genetics 186: 997-1012.

8. Kozlowska JL, Ahmad AR, Jahesh E, Cutter AD (2012) Genetic variation for post-zygotic reproductive isolation between *Caenorhabditis briggsae* and *Caenorhabditis* sp. 9. Evolution 66: 1180-1195.

9. Yan C, Bi Y, Yin D, Zhao Z (2012) A method for rapid and simultaneous mapping of genetic loci and introgression sizes in nematode species. PLoS ONE 7: e43770.

10. Lo T-W, Pickle CS, Lin S, Ralston EJ, Gurling M, Schartner CM, Bian Q, Doudna JA, Meyer BJ (2013) Heritable genome editing using TALENs and CRISPR/Cas9 to engineer precise insertions and deletions in evolutionarily diverse nematode species. Genetics 395: 331-348.

11. Jovelin R (2013) Pleiotropic constraints, expression level, and the evolution of miRNA sequences. Journal of Molecular Evolution 77: 206-220.

12. Lin KT-H, Broitman-Maduro G, Hung WWK, Cervantes S, Maduro MF (2009) Knockdown of SKN-1 and the Wnt effector TCF/POP-1 reveals differences in endomesoderm specification in *C. briggsae* as compared with *C. elegans*. Developmental Biology 325: 296-306.

13. Gimond C, Jovelin R, Han S, Ferrari C, Cutter AD, Braendle C (2013) Outbreeding depression with low genetic variation in selfing *Caenorhabditis* nematodes. Evolution 67: 3087-3101.

14. Hodgkin J, Felix M-A, Clark Laura C, Stroud D, Gravato-Nobre Maria J (2013) Two *Leucobacter* strains exert complementary virulence on *Caenorhabditis* including death by worm-star formation. Current Biology 23: 2157-2161.

15. Dey A, Jeon Y, Wang G-X, Cutter AD (2012) Global population genetic structure of *Caenorhabditis remanei* reveals incipient speciation. Genetics 191: 1257-1269.

16. Jovelin R, Cutter AD (2013) Fine-scale signatures of molecular evolution reconcile models of indel-associated mutation. Genome Biology and Evolution 5: 978-986.

17. Kiontke K, Sudhaus W (2006) Ecology of *Caenorhabditis* species. In: The *C. elegans* Research Community, editor. Wormbook. (January 09, 2006) ed. doi/10.1895/wormbook.1.37.1: <http://www.wormbook.org>.

18. Kiontke K, Barriere A, Kolotuev I, Podbilewicz B, Sommer R, Fitch DH, Félix M-A (2007) Trends, stasis, and drift in the evolution of nematode vulva development. Current Biology 17: 1925-1937.

19. Kiontke K, Félix M-A, Ailion M, Rockman M, Braendle C, Pénigault J-B, Fitch D (2011) A phylogeny and molecular barcodes for *Caenorhabditis*, with numerous new species from rotting fruits. BMC Evolutionary Biology 11: 339.

20. Félix M-A, Jovelin R, Ferrari C, Han S, Cho YR, Andersen EC, Cutter AD, Braendle C (2013) Species richness, distribution and genetic diversity of *Caenorhabditis* nematodes in a remote tropical rainforest. BMC Evolutionary Biology 13: 10.

21. Winston WM, Sutherlin M, Wright AJ, Feinberg EH, Hunter CP (2007) *Caenorhabditis elegans* SID-2 is required for environmental RNA interference. Proceedings of the National Academy of Sciences USA 104: 10565-10570.

22. Baldi C, Viviano J, Ellis RE (2011) A bias caused by ectopic development produces sexually dimorphic sperm in nematodes. Current Biology 21: 1416-1420.

23. Nuez I, Félix MA (2012) Evolution of susceptibility to ingested double-stranded RNAs in *Caenorhabditis* nematodes. PLoS ONE 7: e29811.

24. Pénigault J-B, Félix M-A (2011) Evolution of a system sensitive to stochastic noise: P3.p cell fate in *Caenorhabditis*. Developmental Biology 357: 419-427.

25. Raboin MJ, Timko AF, Howe DK, Félix M-A, Denver DR (2010) Evolution of *Caenorhabditis* mitochondrial genome pseudogenes and *C. briggsae* natural isolates. Molecular Biology and Evolution 27: 1087-1096.

26. Brauchle M, Kiontke K, MacMenamin P, Fitch DHA, Piano F (2009) Evolution of early embryogenesis in rhabditid nematodes. Developmental Biology 335: 253-262.

27. Blaxter M, Kumar S, Kaur G, Koutsovoulos G, Elsworth B (2012) Genomics and transcriptomics across the diversity of the Nematoda. Parasite Immunology 34: 108-120.

28. Sudhaus W (2011) Phylogenetic systematisation and catalogue of paraphyletic "Rhabditidae" (Secernentea, Nematoda). Journal of Nematode Morphology and Systematics 14: 113-178.

29. Schneider W (1937) Freilebende Nematoden der Deutschen Limnologischen Sundaexpedition nach Sumatra, Java und Bali. Archiv für Hydrobiologie 15: 30-108.

30. Tsuda K, Futai K (1999) Description of *Caenorhabditis auriculariae* n. sp. (Nematoda: Rhabditida) from fruiting bodies of *Auricularia polytricha*. Japanese Journal of Nematology 29: 18-23.

31. Schmidt GD, Kuntz RE (1972) *Caenorhabditis avicola* sp. n. (Rhabditidae) found in a bird from Taiwan. Proceedings of the Helminthological Society of Washington 39: 189-191.

32. Kreis HA (1964) Beiträge zur Kenntnis parasitischer Nematoden 23. Ein neuer Nematode aus dem äuβeren Gehörgang von Zeburindern in Ostafrika, *Rhabditis bovis* n. sp. (Rhabditidoidea; Rhabditidae). Schweizer Archiv für Tierheilkunde 106: 372-378.

33. Kreis HA, Faust EC (1933) Two new species of *Rhabditis* (*Rhabditis macrocerca* and *R. clavopapillata*) associated with dogs and monkeys in experimental *Strongyloides* studies. Transactions of the American Microscopical Society 52: 162-172.

34. Baird SE (2001) Strain-specific variation in the pattern of caudal papillae in *Caenorhabditis briggsae* (Nematoda: Rhabditidae); implications for species identification. Nematology 3: 373-376.

35. Völk J (1950) Die Nematoden der Regenwürmer und aasbesuchenden Käfer. Zool Jb Syst 79: 1-70.

36. Yokoo T, Okabe K (1968) Two new species of genus *Rhabditis* (Nematoda: Rhabditidae) found in the intermediate host of *Schistosoma japonica*, *Oncomelania hupensis nosophora* and *Oncomelania hupensis formosana*. Agricultural Bulletin of Saga University 25: 69-78.

37. Shinohara T (1960) [Studies on *Rhabditis* (Nematoda, Rhabditidae)] (In Japanese). Journal of the Kurume Medical Association 23: 2777-2819.

38. Scheiber SH (1880) Ein Fall von mikroskopisch kleinen Rundwürmern – *Rhabditis genitalis* – im Urin einer Kranken. Archiv für Pathologische Anatomie und Physiologie und für klinische Medicin 82: 161-175.

39. Maupas E (1900) Modes et formes de reproduction des nématodes. Archives de Zoologie Expérimentale et Générale 8: 463-624.

40. Kiontke K (1997) Description of *Rhabditis* (*Caenorhabditis*) *drosophilae* n. sp. and *R*. (*C*.) *sonorae* n. sp. (Nematoda: Rhabditida) from saguaro cactus rot in Arizona. Fundamental and Applied Nematology 20: 305-315.
